# The state of global research on poverty after SDG declaration

**DOI:** 10.1002/puh2.110

**Published:** 2023-07-17

**Authors:** Kehinde Kazeem Kanmodi, Jimoh Amzat, Lawrence Achilles Nnyanzi

**Affiliations:** ^1^ School of Health and Life Sciences Teesside University Middlesbrough UK; ^2^ Faculty of Dentistry University of Puthisastra Phnom Penh Cambodia; ^3^ Cephas Health Research Initiative Inc Ibadan Nigeria; ^4^ School of Dentistry University of Rwanda Kigali Rwanda; ^5^ Department of Sociology Usmanu Danfodiyo University Sokoto Nigeria; ^6^ Department of Sociology University of Johannesburg Johannesburg South Africa; ^7^ School of Public Health King Ceasor University Kampala Uganda

**Keywords:** bibliometric analysis, global development, global health, inequality, poverty, research capacity, review, SDG, Sustainable Development Goals, United Nations

## Abstract

**Background:**

Poverty is a significant global problem which can hinder the attainment of the United Nations’ (UN) Sustainable Development Goals (SDGs). Poverty reduction or elimination requires adequate scientific or research contributions, creating socio‐scientific frames that could inform relevant stakeholders in policy planning, implementation and evaluation. This study aims to review the state of global research on poverty (2016 and upwards), since the UN's declaration of SDGs in 2015.

**Methods:**

This study adopted a bibliometric review design. On 31 May 2022, a systematic SCOPUS‐based search was conducted to retrieve all journal papers published on poverty (2016 till 31 May 2022). The bibliometric data of the retrieved papers were analysed using Microsoft Excel and VOSviewer software.

**Result:**

A total of 15,143 journal papers on poverty were retrieved, of which 91.2% were published in English, whereas slightly more than half (52.9%) were published in the social sciences. Although all regions of the world sourced journal papers on poverty, however, countries from the Global North, particularly the USA and the United Kingdom, dominated other countries in terms of authorship, funding and institutional affiliations. Among Global South countries, China leads in terms of authorship, funding and institutional affiliations. South America and Africa contributed the smallest volume of journal papers on poverty.

**Conclusion:**

There exist global inequalities in research productivity on poverty. The global poverty problem skews to the Global South, but the scientific contributions flow from the Global North. There is a need to narrow the existing inequality gaps in the research productivity on poverty through North–South synergetic research collaborations.

## INTRODUCTION

Poverty remains a significant global problem; it is multi‐dimensional with multifaceted consequences. Poverty is a condition of inadequate access to basic human needs, including food, clean water, clothing and shelter. Poverty, intensified by marginalisation and exclusion, accounts for all forms of deprivations of economic resources, education, healthcare, food, employment and recreation [1]. Hence, ‘Zero Poverty’ is spelt out as a goal by the United Nations (UN) as Sustainable Development Goal One (SDG1) with five targets to eradicate extreme poverty by lifting women, children and other marginalised groups out of poverty through efficient social protection initiatives and non‐discrimination in the allocation of economic resources.

Up to 10% of the global population lives in extreme poverty, that is below the poverty line, that is less than US$1.9 a day [2]. Poverty is not evenly distributed. Almost 80% of those living in extreme poverty are in South Asia and sub‐Saharan Africa – the trio of India, Nigeria and the Democratic Republic of Congo have the highest poverty incidence [1]. The human suffering as a result of poverty is unimaginable and affects all facets of life.

Poverty is a very serious problematic issue because it is responsible for numerous social problems, including population pressure, crime, high disease burden, food insecurity and human trafficking, among others [3]. Poverty can reproduce itself by generating consequences that further aggravate itself. For instance, poverty leads to inadequate access to education, which exacerbates poverty [4]. Hence, the vicious cycle of poverty is about the constellation of multiple factors acting and reacting to sustain poverty.

Research has indicated that poverty, a central theme in development discourse, is a disaster with severe adverse effects on all development indicators and other SDGs [5, 6]. For instance, poverty is a cause of malnutrition, poor access to healthcare and environmental degradation [7]. It is challenging for most low‐income communities to achieve universal healthcare due to poverty and inadequate social protection (including health insurance) [8]. Hence, the disease burden is very high, resulting in poor health outcomes (this is related to SDG3) in most low‐income countries [8]. As a result of poverty, an erosive lifestyle prevails, exposing people to increased morbidity and mortality. Critical life opportunities elude the poor because of general social inequity. There is a tendency for the poor to remain poor because of the (harmful) coping strategies they adopt which create a culture of poverty; this includes beliefs and practices that intensify the impoverished conditions.

Poverty is linked to environmental degradation and climate‐induced disasters such as flooding and drought [9]. Poverty drives people into the slums with inadequate water, sanitation and hygiene (this is related to SDG6). People cut down trees for firewood used in cooking, leading to forest depletion (this is related to SDG7), giving room for flood risk [10]. It was observed that the adverse effects of weather shocks include flood, drought, decrease in consumption and increase in extreme poverty, across sub‐Saharan Africa [11]. Smallholder farmers are more vulnerable to the risk of climatic variability with significant cascading effects, such as low productivity, food crisis and poverty. Poverty widens inequality gaps (this is related to SDG9), and it prevents access to quality education (this is related to SDG4). It has a gender dimension as it is disproportionately prevalent among women (this is related to SGD5). It is inimical to responsible consumption (this is related to SDG12), decent employment (this is related to SDG8), sustainable cities (this is related to SDG11) and climate action (this is related to SDG13).

Poverty accounts for deplorable practices with adverse effects on living, whether on land (this is related to SDG15) or below water (this is related to SDG14). Poverty is a significant indicator of weak and non‐inclusive institutions and violence worldwide (this is related to SDG16). Poverty is connected to all the development goals; therefore, reducing poverty through effective partnerships (this is related to SDG17) can enhance the attainment of all the other goals. Although there has been some progress in reducing global poverty over the years, the quadruple threat of the global pandemic (especially the coronavirus of 2019), war/conflict, climate change and weak political resolve have exacerbated the situation [12, 13]. Hence, ending extreme poverty by 2030 might not be feasible unless there are renewed global efforts (this is related to SDG17) to tackle its prevailing threats.

The preceding arguments have highlighted the connection between poverty reduction and development goals. Despite the significance of poverty reduction/eradication, one critical challenge is sustaining the discourse on poverty in a position of the utmost priority, especially by the scientific community. The goal of reducing or eradicating poverty, like other goals, requires adequate scientific or research contributions, which create framings (i.e. socio‐scientific construction) and draw the attention of relevant stakeholders. Research often provides evidence which is required in policy strategies against poverty [14]. The fundamental question here is whether poverty has attracted a deserving voice from the scientific community in various global regions. Pertinently, this fundamental question can be answered using a bibliometric review approach.

A bibliometric review is a research approach that analyses the frequencies, patterns and impacts of research contributions made by researchers, journals, organisations, countries and continents on a research topic [15, 16, 17]. This approach identifies the priorities on a research topic and provides researchers with crucial information on the research growth and future directions in an academic discipline [18, 19, 20, 21, 22, 23, 24, 25].

This study – a bibliometric review – seeks to identify the major contributors, themes and trends of poverty research using citation scooping and analysis and obtain information about scientific productivity and the geographical distribution of research and scholarly contributions to poverty [18, 19, 20, 21, 22, 23, 24, 25]. This bibliometric review will also visualise the growth of research productivity and progress concerning poverty from 2016 – the year after SDGs were instituted – to date and identify the research priorities according to the topic of poverty.

## METHODS

### Study design

This study was a bibliometric review of global research on poverty. The methodological approach used for this review was based on the guidelines proposed by Donthu et al. [26], and the approach has been adopted in several bibliometric reviews [20, 21, 22, 23, 24, 25].

### Literature search

On 31 May 2022, an advanced filtered search of peer‐reviewed journal papers from 2016 to date on poverty on SCOPUS (Box S1). Out of the existing databases, this study used the SCOPUS database for this search because it is the biggest and most comprehensive database for research publications [27]. SCOPUS has a pre‐developed and highly comprehensive search string for scooping out research publications on poverty (see Box S1). SCOPUS is also user‐friendly, and it has bibliometric review‐friendly operating commands [20–25, 28].

The filtered search generated 15,143 journal papers which were all included for bibliometric data extraction. The following bibliometric data were extracted:

- Citation information: authors’ names, journal paper title, journal title, journal paper type, publication year and volume, issue number, page numbers and citation counts
- Bibliographical information: institutional and country affiliations and language of publication
- Index keywords
- Details of funding sponsor
- *h*‐Index (of authors, journal papers, journals, institutions and countries). The *h*‐index is a measure of influence, and it refers to the total number of h papers cited at least h times [26]
- CiteScore 2021 of the relevant journals. CiteScore is a ranking used by SCOPUS to determine the level of impact of a journal. CiteScore is calculated based on the total citations (TCs) accumulated over 4 years. For CiteScore 2021, its formula is: $CiteScore\;2020\;=\frac{\begin{array}{c} Number\;of\;citations\;received\;in\;2018-2021\;to\;5\;published\;document\;\\ types\;(articles,\;etc.)\;by\;a\;journal\;in\;the\;same\;4\;years \end{array}}{\begin{array}{c} Total\;number\;of\;documents\;indexed\;in\;SCOPUS\;and\;\\ published\;in\;2018-2021\; \end{array}}\;$

These bibliometric data were exported from SCOPUS in .csv format; this was done on the day of the literature search (31 May 2022) to avoid the biases and discrepancies that may occur because of the SCOPUS database update [21, 22, 23, 24, 25, 28].

### Data analysis

Two software were used to analyse the extracted datasets – Microsoft Excel version 2021 and VOSviewer version 1.6.18. The Microsoft Excel version 2021 software was used for the performance analysis of the bibliometric metrics of the top 10 authors, journals, institutions and countries/territories sourcing/funding journal papers on poverty [21, 22, 23, 24, 25, 26]. These metrics included total publications (TPs), TCs and *h*‐index. Additionally, these top 10 entities were ranked based on the volume of outputs on poverty. The findings obtained from this analysis were presented in texts, geographical maps, charts and frequency tables.

The VOSviewer version 1.6.18 software was used for the scientific mapping of co‐authorships and index keyword co‐occurrences of the top 2000 most‐cited journal papers on poverty through network visualisations [26, 28]. The co‐authorship analysis was used to explore the interactions among these authors, institutions and countries [21, 24, 25, 26]. The analysis included the interconnection of those authors/institutions/countries with at least five journal papers and at least one citation. The index keyword co‐occurrence analysis was used to examine the relationship among the index keywords used in those papers [21, 24, 25, 26]; only those index keywords occurring in at least 10 papers were used in the analysis.

## RESULTS

### Publication attributes

The 15,143 journal papers on poverty were published in 36 languages; however, the top 10 languages were European and Chinese. The three most common languages of publication were English (first), Spanish (second) and Chinese (third). Moreover, the publications in these three languages had the top 10 TCs and *h*‐indexes (Table 1).

**TABLE 1:** Top 10 languages of journal papers on poverty.

Rank	Language	Total publications^a^	Total citations	*h*‐Index
1st	English	14,064	89,582	80
2nd	Spanish	376	514	11
3rd	Chinese	239	819	13
4th	Portuguese	164	632	11
5th	Russian	140	134	5
6th	French	126	162	5
7th	German	54	40	3
8th	Italian	48	53	4
9th	Croatian	10	9	2
10th	Ukranian	9	8	2

^a^ Some papers may be published/translated in more than one language.

**FIGURE 1:**
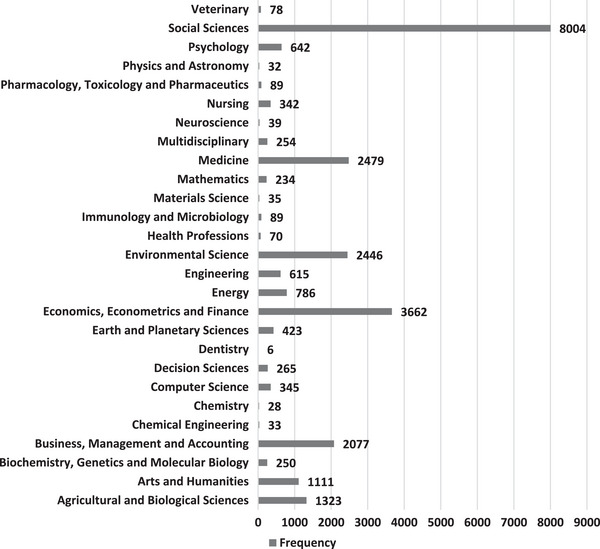
Distribution of journal papers on poverty by subject area.

Figure 1 shows the distribution of these 15,143 journal papers by subject area. More than half (52.9%) of these publications were in the social sciences, whereas the fewest (<0.001%) were in dentistry.

**FIGURE 2:**
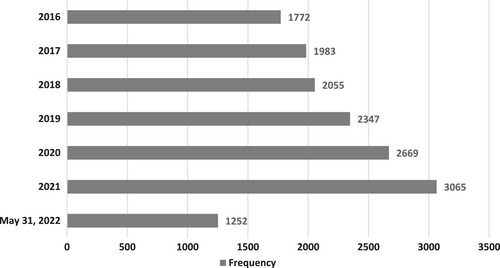
Distribution of journal papers on poverty by year.

Figure 2 shows the distribution, by year, of journal papers on poverty. In 2016 alone, a total of 1772 journal papers were published, and from 2016 to date, there has been a steady growth in the volume of papers within the scope of the goal.

Table 2 shows the distribution, by document type, of journal papers on poverty. The majority (91.0%) of these papers were articles, 6.3% were reviews and the rest were other forms of journal papers. Articles had the highest TCs (89,583) and *h*‐index (80) compared to other document types.

**TABLE 2:** Distribution of journal papers on poverty by document type.

Rank^a^	Document type (*n* = 15,143)	Total publications (%)	Total citations	*h*‐Index
1st	Article	13,780 (91.0)	89,583	80
2nd	Review	949 (6.3)	9731	46
3rd	Note	127 (0.8)	469	11
4th	Conference paper	98 (0.6)	424	11
5th	Editorial	79 (0.5)	523	11
6th	Erratum	38 (0.3)	4	1
7th	Letter	32 (0.2)	173	9
8th	Short survey	17 (0.1)	258	8
9th	Data paper	11 (0.1)	20	3
10th	Undefined	9 (<0.1)	No data	No data
11th	Retracted	3 (<0.1)	No data	No data

^a^ Ranking was based on total number of publications per category.

### Top 10 analyses of the journal papers

**FIGURE 3:**
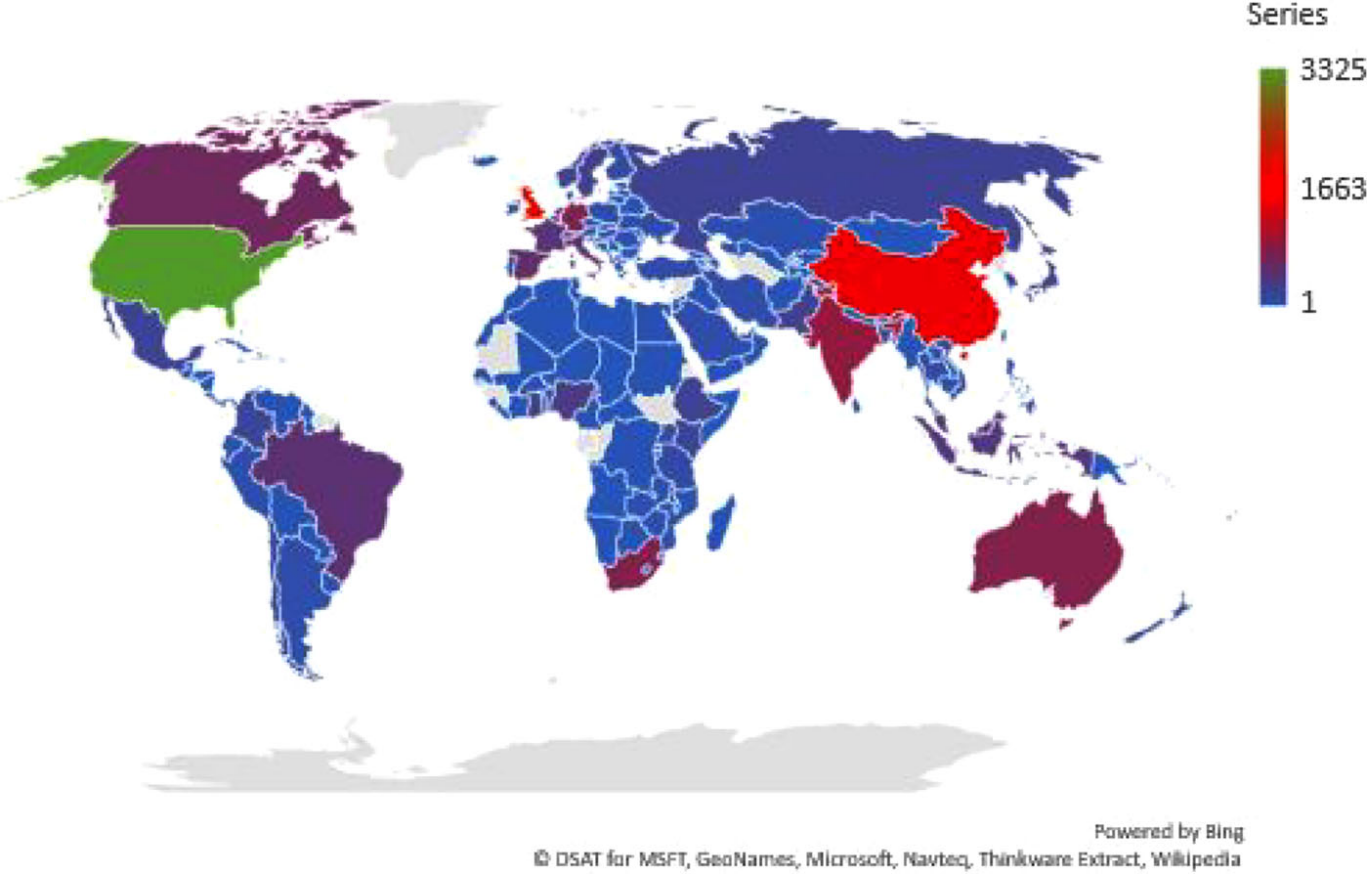
Geographical mapping of global contributions on poverty.

A total of 160 territories and countries sourced these journal papers. Figure 3 shows the geographical mapping of the contributions, in terms of the volume of outputs, of each country/territory. The USA (*n* = 3325) and the United Kingdom (*n* = 1856) were the countries with the highest volume of journal papers.

Table 3 shows the top 10 countries with the highest number of journal papers on poverty. Four tenth of these countries were European, whereas one tenth was African. Among these top 10 countries, the United States (rank = 1st) had the highest volume of papers with TP, TC and *h*‐index at 3325, 33,246 and 66, respectively.

**TABLE 3:** Top 10 countries with the highest volume of journal papers on poverty.

Rank^a^	Country	Continent	Total publications^b^	Total citations	*h*‐Index
1st	United States	North America	3325	33,246	66
2nd	United Kingdom	Europe	1856	19,964	57
3rd	China	Asia	1506	11,294	45
4th	India	Asia	873	3883	26
5th	Australia	Australia	763	7615	39
6th	South Africa	Africa	745	5054	30
7th	Germany	Europe	740	7090	38
8th	Canada	North America	627	5957	32
9th	Spain	Europe	516	2970	24
10th	Italy	Europe	474	4111	32

^a^ Ranking was based on total number of publications per category.

^b^ A journal paper can be affiliated to two or more countries.

The total number of institutions sourcing these journal papers could not be ascertained due to the inconsistencies in the affiliation names of authors; however, the top 10 institutions were identified. Table 4 shows the top 10 institutions with the highest volume of journal papers on poverty. Seven tenth of these institutions were universities, of which only one of them is privately owned. Aside from the institutions jointly owned by the UN member states (inclusive of African countries), the University of Cape Town was the only African institution that made the top 10 list. The Chinese Academy of Sciences, owned by the Chinese Government, had the highest number of papers (TP = 190 papers), citations (TC = 2924) and *h*‐index (*h*‐index = 28) concerning poverty.

**TABLE 4:** Top 10 institutions with the highest volume of journal papers on poverty.

Rank^a^	Institution	Owner	Total publications^b^	Total citations	*h*‐Index
1st	Chinese Academy of Sciences	Chinese Government	190	2924	28
2nd	University of Oxford	UK Government	152	1005	16
3rd	The World Bank	United Nations Member States	151	1655	21
4th	London School of Hygiene and Tropical Medicine	UK Government	130	961	16
5th	London School of Economics and Political Science	UK Government	116	957	16
6th	University of Cape Town	South African Government	113	1343	20
7th	University College London	UK Government	110	845	15
8th	Institute of Geographical Sciences and Natural Resources Research	Chinese Government	108	1995	24
9th	Columbia University	Private	106	1300	14
10th	The Australian National University	Australian Government	99	855	15

^a^ Ranking was based on the total number of publications per category.

^b^ A journal publication can be affiliated to two or more institutions; UK – United Kingdom; US – United States.

Table 5 shows the top 10 funders of journal papers on poverty. The US and UK governments, each, solely owned three tenth of these funding organisations. However, the funding organisation having the highest volume of journal papers (TP = 535), citations (TC = 4949) and *h*‐index (*h*‐index = 32) was the National Natural Science Foundation of China – an institution solely owned by the Chinese government.

**TABLE 5:** Top 10 most productive funders of journal papers on poverty.

Rank^a^	Funding organisations	Owner	Total publications^b^	Total citations	*h*‐Index
1st	National Natural Science Foundation of China	Chinese Government	535	4949	32
2nd	National Institutes of Health	US Government	272	2012	12
3rd	European Commission	European Union Member States	243	2458	26
4th	Economic and Social Research Council	UK Government	221	2279	24
5th	UK Research and Innovation	UK Government	199	2168	24
6th	U.S. Department of Health and Human Services	US Government	170	1197	17
7th	World Bank Group	United Nations Member States	138	1005	15
8th	National Science Foundation	US Government	137	1672	20
9th	Ministry of Education of the People's Republic of China	Chinese Government	136	664	14
10th	Department for International Development	UK Government	132	1226	19

^a^ Ranking was based on total number of publications per category.

^b^ A journal publication can be affiliated to two or more funders; UK – United Kingdom; US – United States.

Table 6 shows the list of the top 10 journals with the highest volume of papers on poverty. In terms of volume of outputs, *Sustainability* (TP = 307), *World Development* (TP = 297) and *Development in Practice* (TP = 212) were the top three sources of journal papers on poverty. Among these top three journals, *World Development* had the highest number of citations (TC = 5309), *h*‐index (*h*‐index = 38) and CiteScore 2021 (*n* = 9.4).

**TABLE 6:** Top 10 journals with the highest volume of journal papers on poverty.

Rank^a^	Journals	Publisher (headquarters)	CiteScore 2021	Total publications	Total citations	*h*‐Index
1st	Sustainability	MDPI (Switzerland)	5.0	307	2207	21
2nd	World Development	Elsevier (Netherlands)	9.4	297	5309	38
3rd	Development In Practice	Taylor & Francis (UK)	2.1	212	833	13
4th	PLoS One	Public Library of Science (USA)	5.6	131	1081	18
5th	Social Indicators Research	Springer Nature (Germany)	4.7	127	1004	17
6th	International Journal of Environmental Research and Public Health	MDPI (Switzerland)	4.5	114	510	11
7th	Development Policy Review	Wiley‐Blackwell (USA)	3.3	109	469	11
8th	Journal of Development Studies	Taylor & Francis (UK)	4.1	97	497	12
9th	Journal of International Development	Wiley‐Blackwell (USA)	3.1	93	505	12
10th	International Journal of Social Economics	Emerald (UK)	2.3	86	362	10

Abbreviations: MDPI, Multidisciplinary Digital Publishing Institute; USA, United States of America; UK, United Kingdom.

^a^ Ranking was based on total number of publications per category.

Table S1 shows the top 10 (most prolific) authors with the highest volume of journal papers on poverty. Based on the volume of outputs, the top three authors were Mersland R (rank = 1st; TP = 24), Gnangnon SK (rank = 1st; TP = 24) and Mia MA (rank = 3rd; TP = 23). However, none of these top three authors was among the top three with the highest *h*‐index – Liu Y (rank = 4th; TC = 951; *h*‐index = 13), Lönnroth K (rank = 7th; TC = 322; *h*‐index = 10) and Shuai C (rank = 5th; TC = 170; *h*‐index = 9). Notably, only 1 of the top 10 authors was currently/most recently affiliated with an institution in Africa, whereas the majority (50%) were affiliated with institutions in Europe. China and the United States have two authors each on the list.

**FIGURE 4:**
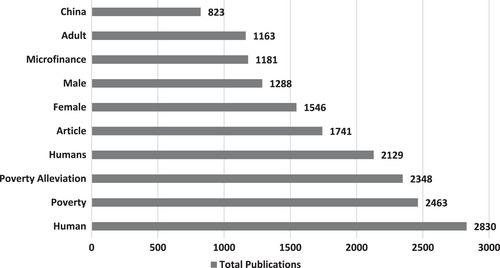
Top 10 keywords used in journal papers on poverty.

Figure 4 shows the top 10 keywords used in the journal papers on poverty. The top three keywords were human (*n* = 2830), poverty (*n* = 2463) and poverty alleviation (*n* = 2348).

China was the only country found in the top 10 keywords used in those papers. Moreover, more papers used ‘female’ (*n* = 1546) than ‘male’ (*n* = 1288) as a keyword in those papers.

### Network visualisations

The findings obtained from the network visualisation analysis of the top 2000 most cited journal papers on poverty are shown in the subsequent figures.

#### Co‐authorship profile of publications

**FIGURE 5:**
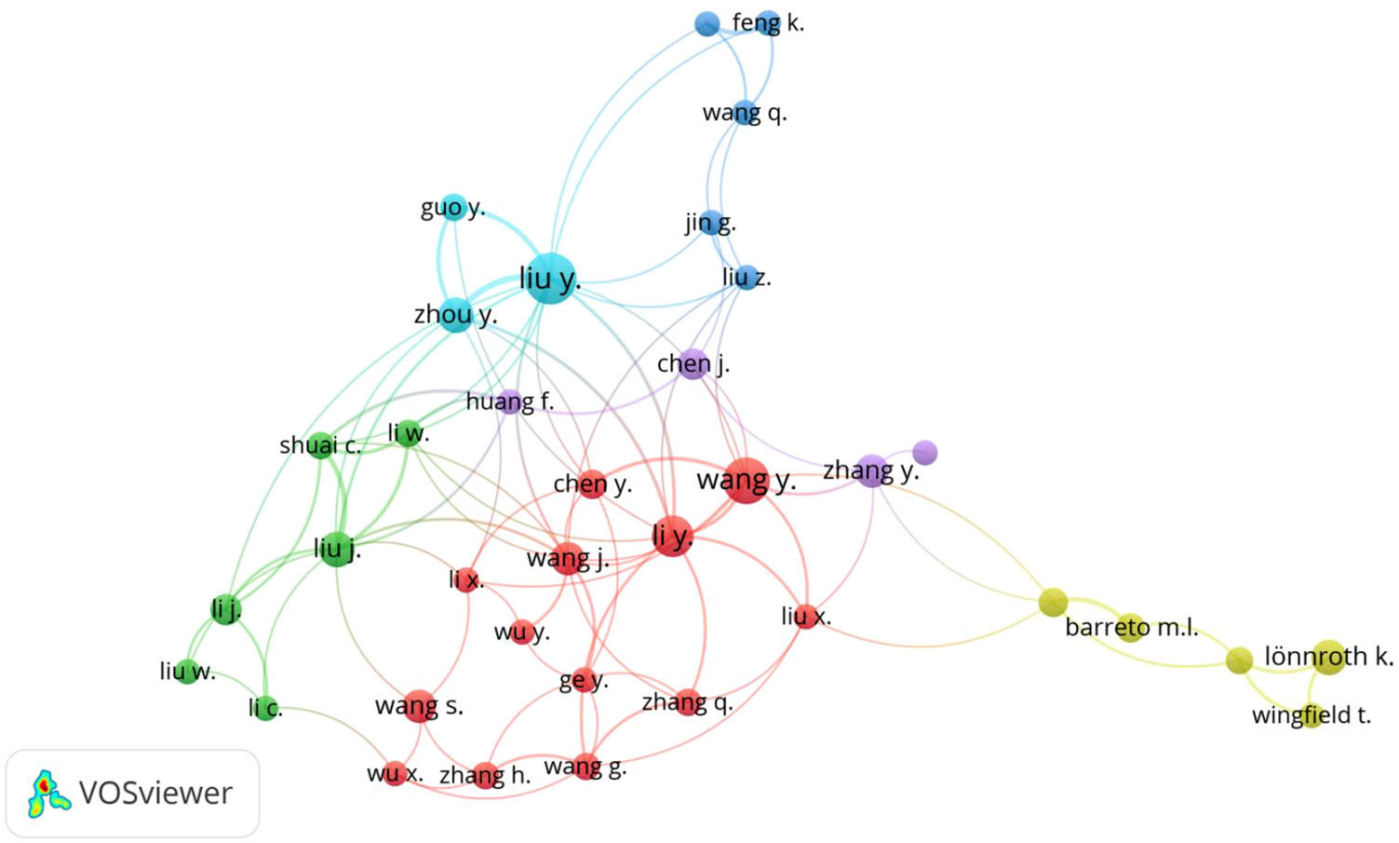
Network visualisation of co‐authorship among authors.

A total of 7177 researchers authored the top 2000 most cited journal papers on poverty. Out of these 7177 authors, only 53 had at least 5 papers each and at least 1 citation. Of these 53 authors, only 36 were interconnected and were included for network visualisation (Figure 5). The total link strength (TLS) of each of these 36 authors was within the range of 1–29. Liu Y (TLS = 29) had the highest TLS. Six clusters were identified, each with its colour. The cluster with the biggest network (based on cumulative TLS) is depicted in red – this cluster is made up of 13 authors (Chen Y, Ge Y, Li X, Li Y, Liu X, Wang G, Wang J, Wang S, Wang Y, Wu X, Wu Y, Zhang H and Zhang Q), of which Li Y (TLS of 19) had the highest TLS among them. Impressively, none of the authors in the red cluster – the cluster with the biggest network – made the list of the top 10 prolific authors (Figure 5; Table S1).

The analysis of co‐authorship among institutions could not be done due to the warning raised by the VOSviewer software, during the analysis, that SCOPUS data on organisations may not have been harmonised, and that they may exist in an inconsistent format. As a result, the study did not perform any network visualisation for the institutions.

#### Co‐authorship among countries/territories

**FIGURE 6:**
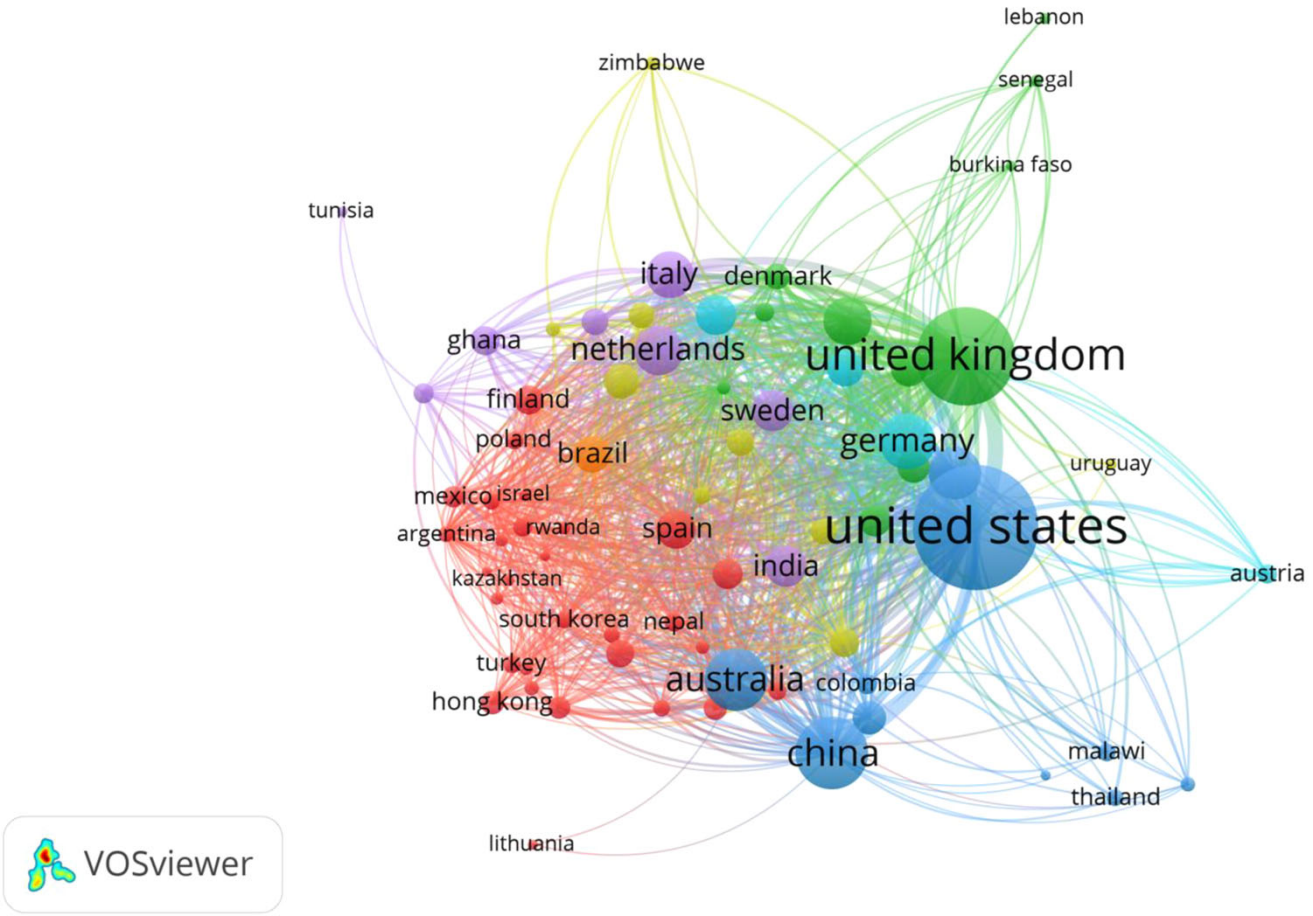
Network visualisation of co‐authorship among countries and territories.

Out of the 172 countries/territories sourcing the top 2000 most cited journal papers on poverty, only 72 sourced at least 5 papers each and had at least 1 citation. Furthermore, all 72 countries/territories were interconnected and included in the network visualisation (Figure 6). The TLS per country/territory ranged from 7 to 664. The United States had the highest TLS (*n* = 604). Seven clusters were identified, each with its colour. The cluster with the most extensive network (based on cumulative TLS) is depicted in red; this cluster is made up of 29 countries (Argentina, Chile, Czech Republic, Egypt, Finland, Greece, Hong Kong, Hungary, Indonesia, Iran, Ireland, Israel, Kazakhstan, Lithuania, Malaysia, Mexico, Nepal, New Zealand, Philippines, Poland, Romania, Russia, Rwanda, Singapore, South Korea, Spain, Taiwan, Turkey and Viet Nam), of which Indonesia (TLS of 119) had the highest TLS.

### Co‐occurrence of index keywords

**FIGURE 7:**
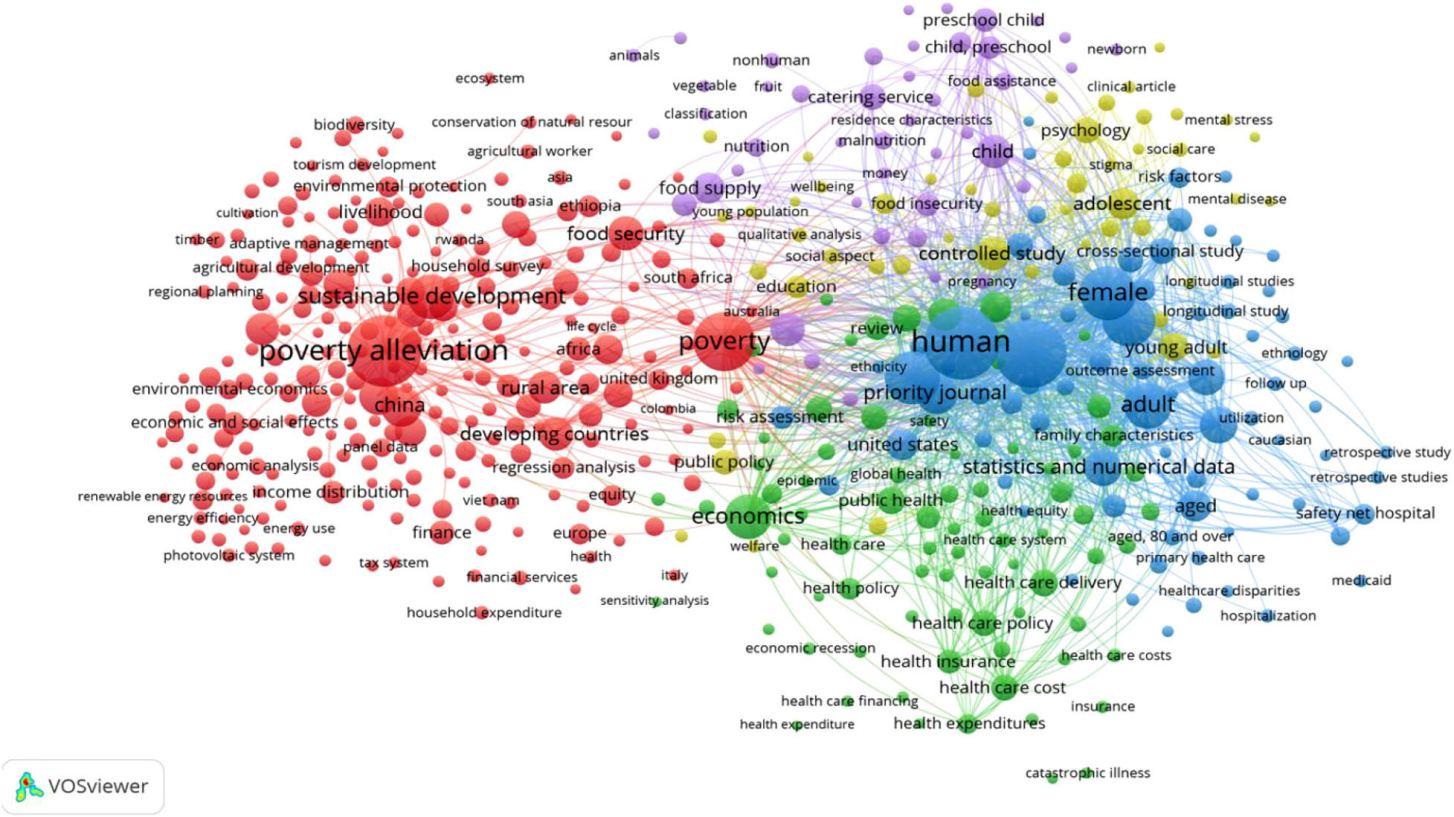
Network visualisation of co‐occurrence among index keywords.

The top 2000 most cited papers on poverty had 6410 index keywords, out of which only 503 occurred in at least 10 papers. Only these 503 keywords were included in the network visualisation (Figure 7). The TLS of each keyword fell within the range of 49–8406. The top three index keywords with the highest TLS were ‘human’ (TLS = 8406), ‘humans’ (TLS = 7883) and ‘female’ (TLS = 5393). Five clusters were identified – each with its colour. The first cluster is the biggest network (based on cumulative TLS) and is depicted in red; this cluster is made up of 252 keywords which are predominantly related to economics, finance, tourism and development. The second cluster (green) is made up of 79 keywords which are related to health sciences. The third cluster (blue) is made up of 77 keywords which are predominantly associated with research design and methodology. The fourth cluster (lemon colour) is made up of 50 items which are mostly related to sociology and social care. The fourth cluster (purple) is made up of 45 keywords which are predominantly associated with maternal and child health.

## DISCUSSION

The study findings highlight an increasing trend in the volume of journal papers on poverty published from 2016 to date. This indicates that the research interest in poverty is progressive. Several reasons may explain this increasing interest, for example the global focus on combating inequality and social injustice, improving education and climate change, all of which are factors influenced by poverty [29, 30, 31]. A similar trend in the observed research productivity rate on poverty has also been observed on some other hot global health and development topics like COVID‐19, psycho‐cardiological diseases and anti‐malarial drug therapy [32, 33, 34]. However, this has not been the case for all research topic areas, as interest in some topics has been static, for example milk fluoridation and fluoridated water–induced cancers [20, 25].

The research interest in poverty is not localised to just a few countries and academic disciplines but diffuse, involving virtually all countries, territories and disciplines. This has not been the case for some other research topics. For example, research interests in community fluoridated water and cancer, milk fluoridation and Qi Gong have been localised to very few countries and disciplines [20, 25, 35]. The global diffuseness in the research on poverty indicates that in all member states of the UN, many researchers and many institutions in different disciplines are keen to investigate poverty‐related problems. However, the volume of research productivity across countries and disciplines was disproportionate. Notably, most (80%) of the top 10 most productive countries sourcing the highest volume of papers on poverty were high‐income countries in the Global North. In contrast, unfortunately, the countries in the Global South and LMICs were less represented. This observation is expected due to the wide funding gap for research in almost all fields between the Global North and South [20, 21, 22, 23, 24, 25]. Pertinently, this finding is consistent with that reported concerning global research output patterns in the fields of technology, clinical medicine, transportation, public health and other indispensable fields needed for sustainable growth and development [36, 37, 38, 39, 40, 41, 42].

The two disciplines with the highest research productivity on poverty were ‘Social Sciences’ and ‘Economics, Econometrics, and Finance’; this may be because poverty is widely seen as a socio‐economic problem and hence getting more focus from researchers in these two disciplines compared to the other disciplines [43]. The emphasis on participatory approaches in studying poverty to elicit real‐life experiences and voices of those that have lived in poverty renders these disciplines more suitable for poverty studies.

Poverty is not only a socio‐economic problem but a problem affecting human health and all other aspects of human and societal development [44, 45]. However, the research productivity on poverty from disciplines, such as ‘Dentistry’, ‘Decision Sciences’, ‘Nursing’ and engineering disciplines was very low. This shows the need to scale up research productivity in these disciplines because research contributions enhance the holistic understanding of the poverty problem, which thus helps in informing evidence‐based strategies needed for eliminating it [46, 47, 48].

From a further examination of the productivity of the top 2000 most influential journal papers (based on the *h*‐index) on poverty, we observed that roughly four tenth (41.8%; 72/172) of the countries sourcing these papers contributed at least five papers each. This may indicate that the journal papers produced by many countries are less influential. The influence of a scientific publication is predominantly determined by its *h*‐index, while the *h*‐index is determined by citation count [26]. The articles that are novel, of high quality and published in a widely understood language and highly visible and impactful journals tend to garner more citations than those that are not. Therefore, to boost the level of influence of the contributions of low‐contributing countries/territories, it is recommended that low‐contributing countries/territories might consider investing more funds and promoting novel research projects on poverty. This is particularly important because poverty influences almost all aspects of society – health, education, living standards, infrastructure, governance and so on [3, 4, 5, 6, 7, 8, 9, 10, 11, 12, 13, 14].

It is also worth noting that those 72 countries having at least 5 papers in the top 2000 most influential journal papers on poverty were interconnected in the network visualisation. This may suggest that inter‐country collaboration produces significant research findings and papers. Therefore, more research partnerships should be encouraged, particularly among high‐income countries and LMICs. In addition to the aforementioned, it is recommended that research partnerships between these countries should be non‐parasitic but synergistic [49, 50]. Approaching research on poverty, by collaborating countries, as equal partners may not only improve the quality of the outputs, but it may also lead to real capacity development [49, 50].

Furthermore, aside from those institutions owned by the member‐states of the UN and the European Commission, the top 10 most productive institutions sourcing or funding journal papers on poverty were based in China, the USA, the United Kingdom, Australia and South Africa. None of the institutions from the South American continent made the list; this suggests relatively lower research productivity in that region. Several South American and African countries ranked atop the list of impoverished countries [51], but their institutions were underrepresented in research productivity on poverty. This may be due to limited resources being invested in research on poverty but also the expertise and capacity to conduct robust research may be a limiting factor in these countries.

Huge investments in research and development are very crucial in poverty eradication [52]. This can be explained using the situational trend of China. Some decades ago, China was regarded as one of the top countries with the largest population of poor people [52]. However, as evidenced by this review's findings, China currently appears among the top 10 keywords used in journal papers on poverty; moreover, the country ranked among the top 10 sources and funders of journal papers on poverty. This shows that huge investments in poverty research are worthwhile.

It is worth noting that no private institution made the list of the top 10 most productive funding sponsors of research projects on poverty. All these top 10 sponsors were owned by national governments, not local, state or provincial governments. With the current magnitude of global poverty‐related challenges, the need for deeper and more active involvement of private sectors, including all arms of government, in sponsoring research projects on poverty cannot be overemphasised [53, 54]. Looking into the past and the present, private sectors have played very crucial and active roles in synergistically enhancing the collective response towards myriads of global development and protection efforts, such as disease outbreak control, malaria eradication, food production and many others [55, 56, 57]. Therefore, more of this should be encouraged in developing the global research capacity on poverty.

There is a Global North–South divide in the distribution of the top 10 most productive and most influential (based on *h*‐index) journals/publishers of papers on poverty: All the top 10 journals/publishers were headquartered in the high‐income Global North countries (the USA, the United Kingdom, the Netherlands, Switzerland and Germany). This is a situation of concern because no country in the Global South hosted any of the headquarters of these journals/publishers. This may, invariably, pose a barrier to access to such journals by researchers from the LMICs.

It is also important to note that these journals predominantly publish their papers in English, yet many researchers in the Global South are not native English speakers [58, 59]. This language barrier may contribute to the existing inequalities in the global research productivity on poverty [49]. Another serious problem is the high fees required for publishing papers in the top 10 leading journals on poverty. Most of the researchers from the Global South have little or no institutional funding and, therefore, could not afford to publish their works in these leading journals [58, 59, 60]; this, therefore, might have reduced their chances of publishing their works in those highly influential journals.

Of the top 10 most productive researchers on poverty, only 4 were from the institutions in the Global South (2 researchers from China, 1 from Malaysia and another from South Africa). At the same time, the rest were researchers from the Global North – the USA, Switzerland, Sweden, France and the United Kingdom. The unequal distribution in this list may be due to the lower amount of resources provided for scholarly research funding and promotion in the Global South [49, 59, 60]. Researchers from China, Malaysia and South Africa made the list of the top 10 most productive authors. Although these three countries are in the Global South, they have demonstrated exceptional research scholarship and commitment to poverty research [61, 62].

From the scientific mapping of inter‐personal collaborations among the authors of the top 2000 most cited papers on poverty, we learned that the highly productive research groups (with each author having at least five articles on poverty each) were made up of groups of researchers – the biggest group has 13 researchers. With the current global burden of poverty, there is a need for more influential research clusters across the globe. The study recommends that those researchers and institutions with more expertise in poverty research should facilitate the proliferation of research clusters in less productive institutions and countries. This can be achieved through leadership, mentorship, funding and training [59].

Several keywords were used in the journal papers on poverty. The analysis of keywords indicated that China was the country with the highest research focus on poverty. No country in South America and Africa made the list of the top 10 index keywords of journal papers on poverty. The poverty rate in many countries on these two continents is far higher than that of China [51]. This shows the need for more research focusing on countries with high poverty rates.

This study highlights disparities in the research capacity on poverty by region with more poverty‐stricken regions demonstrating poorer research capacity compared to richer regions. There are deep‐seated challenges that may explain this phenomenon, including the lack of resources, expertise and systems. These trends can only lead to a vicious cycle characterised by worse poverty effects, low productivity and under‐development. Something needs to be done to reverse these trends; it is recommended that developing global research capacity on poverty especially for countries with the lowest outputs should be prioritised. There is an urgent need to ring‐fence funding for research, scholarship and commitment to poverty research in these countries. Research focusing on poverty should be scaled up and translated into real‐life interventions for the benefit of populations.

### Study limitations

This study presents limitations. First, this study used only one research database (SCOPUS) because of the difficulty of synchronising the data obtained from multiple databases for analysis on the VOSviewer [21, 24, 25]. There is a possibility that some journal papers not indexed in SCOPUS might have been excluded. However, this remains a robust study using a SCOPUS‐developed search string specific for poverty to scoop out all relevant papers.

Second, in the scientific mapping of researchers and countries, the study relied on only the top 2000 cited papers. As a result, we might have underestimated the influence of the contributions of researchers and institutions sourcing newly published papers because of their citation count, even if they have been published in high‐impact factor journals.

Third, due to the lack of harmonisation in the nomenclatures of institutions sourcing papers on poverty, we could not provide precise data on the total number of these institutions globally. The study only reported the top 10 most productive institutions to ensure that reliable information was provided.

Notwithstanding these limitations, the findings are crucial clues for an in‐depth understanding of global research on poverty. The research methodology was based on the guidelines of leading experts in bibliometric analysis [26]. The study adopted advanced scientometric tools for the performance analysis and scientific mapping of bibliometric metrics of interest [21, 24, 25].

## CONCLUSION

This study reviews the global growth of scholarly and research productivity on poverty, after the UN's declaration of SDGs. There has been a volume growth in poverty research, from 2016 to date. Although poverty research contributions cut across all regions, countries from the Global North dominated in terms of scholarship, authorship, funding and institutional affiliations. Of the top 10 countries with the highest number of scholarly papers on poverty, 4 countries are from Europe and just 1 from Africa. This shows that the African continent, which is mostly affected by poverty, lags in inherent evidence‐creation, knowledge production and scientific contribution to understanding and resolving the problem. The gross inequality in global poverty research productivity was pronounced with the challenges of poverty skewing to the Global South, and the scientific contributions flowing from the North. There is a need to narrow the existing inequality gaps in the research productivity on poverty between the Global North and South through synergetic collaborations.

## AUTHOR CONTRIBUTIONS

*Conceptualization; data curation; formal analysis; funding acquisition; investigation; methodology; project administration; resources; software; validation; roles/writing – original draft; writing – review and editing*: Kehinde Kazeem Kanmodi. *Conceptualization; project administration; resources; validation; roles/writing – original draft; writing – review and editing*: Jimoh Amzat. *Writing – review and editing*: Lawrence Achilles Nnyanzi.

## CONFLICT OF INTEREST STATEMENT

The authors declare that there is no conflicts of interest that could be perceived as prejudicing the impartiality of the research reported.

## FUNDING INFORMATION

This study was self‐funded.

## Supporting information

Supporting Information

## Data Availability

Data sharing is not applicable to this article as no new data were created or analysed in this study.
