# Supplementary material for: The state of global research on poverty after SDG declaration
Source: Public Health Chall. 2023 Jul 17;2(3):e110. doi: 10.1002/puh2.110 (PMC12039691; doi:10.1002/puh2.110)
Supplement: Supplementary file 1 — Supporting Information [file PUH2-2-e110-s001.docx]

**Box S1. The search string obtained from SCOPUS**

| TITLE-ABS-KEY ( ( {extreme poverty}  OR  {poverty alleviation}  OR  {poverty eradication}  OR  {poverty reduction}  OR  {international poverty line}  OR  ( {financial aid}  AND  {poverty} )  OR  ( {financial aid}  AND  {poor} )  OR  ( {financial aid}  AND  {north-south divide} )  OR  ( {financial development}  AND  {poverty} )  OR  {financial empowerment}  OR  {distributional effect}  OR  {distributional effects}  OR  {child labor}  OR  {child labour}  OR  {development aid}  OR  {social protection}  OR  {social protection system}  OR  ( {social protection}  AND  access )  OR  microfinanc*  OR  micro-financ*  OR  {resilience of the poor}  OR  ( {safety net}  AND  {poor}  OR  {vulnerable} )  OR  ( {economic resource}  AND  access )  OR  ( {economic resources}  AND  access )  OR  {food bank}  OR  {food banks} ) )  AND  ( LIMIT-TO ( PUBYEAR ,  2022 )  OR  LIMIT-TO ( PUBYEAR ,  2021 )  OR  LIMIT-TO ( PUBYEAR ,  2020 )  OR  LIMIT-TO ( PUBYEAR ,  2019 )  OR  LIMIT-TO ( PUBYEAR ,  2018 )  OR  LIMIT-TO ( PUBYEAR ,  2017 )  OR  LIMIT-TO ( PUBYEAR ,  2016 ) )  AND  ( LIMIT-TO ( SRCTYPE ,  "j" ) ) |
| --- |

**Table S1. Top ten authors of journal papers on poverty**

| **Rank*** | **Authors** | **Most Recent/Current Affiliation on SCOPUS** | **Total Publications** | **Total Citations** | **h-index** |
| --- | --- | --- | --- | --- | --- |
| 1^st^ | Mersland, R. | [University of Agder](https://www.scopus.com/affil/profile.uri?afid=60080184), Kristiansand, Norway | 24 | 241 | 8 |
| 1^st^ | Gnangnon, S.K. | World Trade Organization, Geneva, Switzerland | 24 | 60 | 5 |
| 3^rd^ | Mia, M.A. | [Universiti Sains Malaysia](https://www.scopus.com/affil/profile.uri?afid=60000906), Minden, Malaysia | 23 | 168 | 7 |
| 4^th^ | Liu, Y. | [Chinese Academy of Sciences](https://www.scopus.com/affil/profile.uri?afid=60031150), Beijing, China | 19 | 951 | 13 |
| 5^th^ | Shuai, C. | [China University of Geosciences](https://www.scopus.com/affil/profile.uri?afid=60006019), Wuhan, China | 18 | 170 | 9 |
| 5^th^ | Odhiambo, N.M. | [University of South Africa](https://www.scopus.com/affil/profile.uri?afid=60002397), Pretoria, South Africa | 18 | 93 | 6 |
| 7^th^ | Lönnroth, K. | [Karolinska Institutet](https://www.scopus.com/affil/profile.uri?afid=60012311), Stockholm, Sweden | 17 | 322 | 10 |
| 7^th^ | Boccia, D. | [London School of Hygiene and Tropical Medicine](https://www.scopus.com/affil/profile.uri?afid=60031331), London, UK | 17 | 248 | 7 |
| 7^th^ | Ashta, A. | [Burgundy School of Business](https://www.scopus.com/affil/profile.uri?afid=60107783), Dijon, France | 17 | 62 | 5 |
| 10^th^ | Murshid, N.S. | [University at Buffalo, The State University of New York](https://www.scopus.com/affil/profile.uri?afid=60032083), Buffalo, US | 16 | 107 | 6 |
| *Ranking was based on total number of publications per category | | | | | |
